# The outcomes of transobturator tape intervention in the treatment of stress urinary incontinence: Two years’ follow-up

**DOI:** 10.12669/pjms.35.2.603

**Published:** 2019

**Authors:** Bulat Aytek Sik, Hanife Copur, Yilda Arzu ABA

**Affiliations:** 1 *Bulat Aytek Sik, Department of Obstetrics and Gynecology, Istanbul Aydin University, Istanbul, Turkey*; 2 *Hanife Copur, Department of Obstetrics and Gynecology, Istanbul Taksim Research and Education Hospital, Istanbul, Turkey*; 3 *Yilda Arzu ABA, Bandirma Onyedi Eylul University, Health Science Faculty, Balikesir, Turkey*

**Keywords:** Mixed urinary incontinence, Outcomes, Stress urinary incontinence, Transobturator tape

## Abstract

**Objective:**

To evaluate the clinical outcomes and the effects on quality of life of transobturator tape surgery during a 2-year follow-up period in our clinics.

**Methods:**

Eighty-seven patients with stress or mixed urinary incontinence who underwent transobturator tape surgery were included in the study conducted in Istanbul. Taksim. Training. and Research Hospital Gynecology and Obstetrics Clinic, between 2011 and 2013. The patients’ demographic features, incontinence questionnaires, quality of life scores [Incontinence Impact Questionnaire (IIQ-7) and urinary distress inventories (UDI-6)], examination findings, urodynamic results, stress tests, Q tip tests, number of daily pads, ultrasonography, surgery, and cystoscopy results were recorded. Patients were evaluated 23-27 months (mean: 25.40±1.31 months) after their discharge in terms of symptoms, quality of life scores, urodynamic findings, complications, and stress test.

**Results:**

Sixty-three (72.4%) patients had stress incontinence and 24 (27.6%) patients had mixed urinary incontinence. No perioperative complications were observed in our study. After a follow-up period of two years, a significant improvement was detected in the IIQ-7 and UDI-6 questionnaires when compared with the preoperative period. Moreover, the objective cure rate was found as 88.5% (n=77). De novo urge incontinence was obtained in 5.7% (n=5) of patients and was treated with anticholinergics. Perineal pain was present in 3 (3.44%) patients and was treated with analgesics and cold packs. In 2 (2.29%) patients, vaginal mesh erosion was detected and full recovery was achieved with an excision. Urinary retention and bladder perforation was not seen in any patients.

**Conclusion:**

Our study revealed a high objective cure rate, and an improvement in symptoms and quality of life with the transobturator tape operation.

## INTRODUCTION

As one of the most common problems faced in the gynecology/obstetrics practice, stress urinary incontinence (SUI) affects about 4-35% of all women. The successful treatment of this problem, which negatively affects women’s quality of life, is highly important for the patient. Patients usually become self-conscious about sharing this problem with physicians and procrastinate about consulting a physician on the subject. Although conservative methods such as vaginal pessaries and pelvic floor exercises are available, success rates are low. In cases where these methods fail, various surgical treatments are available for treatment.^1^ Presented for the first time in the literature in 2001 as a minimally invasive technique, transobturator tape (TOT) surgery comprises the placement of a sling material under the urethra with a transobturator approach.^2^,^3^ This sling material imitates the role of the pubo-urethral ligament located in this area, whose weakening causes incontinence, and provides sling-like support under the urethra. Heinonen et al. reported the objective and subjective cure rates with a follow-up period of 6.5 years of TOT treatment in SUI as 89% and 83%, respectively.^4^ In the literature, similar rates have been reported in various studies that evaluated the success of TOT treatment in SUI over different follow-up periods.^5^,^6^

We performed the inside-out TOT technique to a patient group with symptoms of stress or mixed urinary incontinence (MUI) and we aimed to present the success rates, and short and long-term complications of our clinic.

## METHODS

This prospective study was conducted by one physician in Istanbul Taksim Training and Research Hospital Gynecology and Obstetrics Clinic, between 2011 and 2013, with 87 patients who had symptoms of urinary incontinence and were diagnosed as having SUI/MUI. This study was approved by the local ethics committee of Istanbul Taksim Training and Research Hospital and written informed consent was obtained from each participant. Patients aged between 37 and 56 years who had SUI and underwent transobturator tape surgery were included in the study. Patients who had undergone surgery previously due to incontinence or pelvic organ prolapsus, those who wished to have children, those with additional gynecopathologies, patients in need of vaginal hysterectomies, and patients with MUI with urge incontinence symptoms were excluded from the study. Baseline assessments included demographic data, medical history, 1-h pad test, and multichannel urodynamic study (UD-2000, Medical Measurement System, Enschede, Netherlands). The quality of life of the patients was evaluated via questionnaires, which included the short form of the Urinary Distress Inventory (UDI-6), and Incontinence Impact Questionnaire (IIQ-7).^7^ Patients were pre- and post-operatively evaluated. SUI diagnosis was evaluated subjectively with a stress test and bladder neck mobility was evaluated with a Q tip test. Urodynamic evaluation was performed with Laborie® Dorado urodynamics equipment. Pre- and postoperative cystometry tests were performed. Urine analysis and urine cultures were requested prior to urodynamic testing and in case of infection, urodynamic evaluation was postponed until after treatment. Cystometric evaluation was performed after measurement of residual urine. The bladder was filled with saline at room temperature at a rate of 50 mL/min. Patients were asked to cough after every 100 mL and urine leakage during or after coughing was detected. Detrusor contractions and related leakages were recorded. The first and severe urine sensations of patients were recorded. Abdominal leak point pressures (ALPL) were used to determine the subtypes of SUI. Low ALPL (0-60 cm H_2_O) was used as a criterion for Type 3 SUI. For Type 2 SUI, ALPL had to be higher (90 cm H_2_O). Detrusor instability was determined by the detection of an increase of 15 cm H_2_O or higher in detrusor pressure. For prophylactic reasons, one g cefazolin was administered prior to surgery and two g of cefazolin was administered after surgery. All patients underwent surgery with spinal anesthesia. In the surgery, TVT-Obturator inside-out (Ethicon®, USA) equipment was used. Cystoscopies were performed after the procedure to check the bladder and urethra. In patients who required it, additional procedures were performed after the placement of the Prolene band and the patient was asked to cough in order to adjust the tension of the band. The durations of TOT, cystoscopies, and additional procedures were recorded. The catheters were removed 24 hours after the surgery and patients with urine residues less than 100 mL were discharged. Intraoperative and postoperative complications were recorded.

Data analyses were performed using the Statistical Package for Social Science (SPSS Inc, Chicago, Illonois, USA) 10.0 program. Continuous data are expressed as mean ± standard deviation (SD) and statistical significance was evaluated using the Wilcoxon signed-rank test. Categorical data are reported as numerical values and percentages. A p value <0.05 was considered as statistically significant.

## RESULTS

In our study, the mean age of the patients was 46.55 ± 5.74 years (min 37, max 56 years). The mean parity of our patients was 4.05 ± 2.09 (min 2, max 11). According to the obstetric history, 82 (94.2%) patients had vaginal births and four patients had cesarean sections. A total of 26 (29.8%) patients were in menopause. The mean body mass index of the patients was 30.9±4.57 kg/m^2^. A total of 63 (72.4%) patients were diagnosed as having SUI and 24 (27.6%) patients had MUI in the urodynamic evaluation. In the physical examination, rectocele was detected in 24 patients, cystocele was detected in 72 patients, and ureterocele was detected in 16 patients (Table-I).

**Table-I:** The demographical properties of patients who received TOT.

Demographical properties of patients	X ± ss
Age	46.55±5.74
Parity	4.05±2.09
Gravida	5.07±3.09
Body Mass Index	30.9±4.57

	*n (%)*

Vaginal birth	76 (94.2)
Sectio	4 (5.8)
Menopause	26 (29.8)
Rectoceles	24 (27.6)
Cystoceles	72 (82.8)
Ureteroceles	16 (18.4)
Hysterectomies	12 (13.8)

Colporrhaphy anterior (CA) was additionally performed on 28 (32%) patients and colporrhaphy posterior (CP) was additionally performed on 4 (4.5%) patients during the operation. TOT procedures alone were performed on 59 (67.8%) patients. After subtracting cystoscopy and additional procedure durations, the mean duration of operation was 10.3 ± 2.74 minutes (min. 6, max. 15 minutes). No intraoperative complications (e.g., hemorrhage, injury to the bladder, urethra or nerves, vascular injury, hematoma) occurred. The mean residual urine volume at the 24^th^ hour following surgery was 41.5 ± 121.9 mL (min. 0, max. 550 mL). With the exception of four patients who had a residual urine volume of 550 mL, all patients were discharged in the 1^st^ postoperative day; the mean duration of stay at the hospital was 1.2 ± 0.89 days. The mean follow-up period of the patients was 25.4 ± 1.31 months (min. 23, max. 27 months).

The mean number of micturition’s during the day and night before the surgery was 6.30 ± 2.83 (min. 3, max. 12) and 1.5 ± 1.40 (min. 0, max. 4), respectively. The mean number of daily pads was 1.5 ± 1.40 (min. 0-max. 4). The mean Q tip test finding was 41.5 5 ± 22.6 (min. 15, max. 80) (Table II).

**Table-II:** Preoperative and postoperative characteristics of the patients.

Preoperative and postoperative characteristics of the patients	Preoperative X ± ss	Postoperative (2nd year) X ± ss	P
Number of micturitions during the day	6.30 ± 2.83	4.34±1.88	0.001^**^
Number of nocturnal urinations	1.40±1.20	1.20±0.56	0.1615
First urinary sensation	178.5±89.05	159.0±39.16	0.0640
Maximum capacity	529.5±80.6	518.75±51.6	0.2968
Residual urinary volume	13.50±3.66	14.09±4.08	0.663
Maximum detrusor pressure	11.60±2.80	10.95±2.95	0.6500
Bladder compliance measurements	63.76±30.79	59.40±11.27	0.2175
IIQ-7 Scores	11.00±2.03	1.25±2.09	0.001^**^
UDI-6 Scores	9.55±3.03	3.15±1.78	0.001^**^
Number of Daily pads	3.55±1.43	0.40±0.60	0.001^**^
Q tip test	41.5 5 ± 22.6	37.75±12.70	0.3078

Despite the postoperative decrease in the number of micturitions during the day and night, the values during the day were statistically significant (p=0.001). According to Q tip test results, bladder neck mobility did not change after surgery (p=0.307). SUI was urodynamically detected in 8 (9.1%) patients and de novo UI was detected in 4 (4.5%) patients after long-term follow-up. The amount of residual urinary volumes during postoperative follow-ups did not change when compared with the preoperative period (p=0.663). No significant change was observed in the first urinary sensation, bladder capacity, bladder compliance or maximum detrusor pressure during the follow-up period. The preoperative and postoperative characteristics of the patients are presented in Table-II.

A significant improvement was seen in the IIQ-7 and UDI-6 questionnaires, which were performed to evaluate the patients’ quality of life when compared with the pre-operative period. In our study, the objective recovery rate of our patients was found as 88.5% (n=77) in their two- year follow-up. De novo urge incontinence was seen in 5.7% (n=5) of patients and was treated with anticholinergics. Urinary retention and bladder perforation was not seen in any patients. Perineal pain was seen in 3 (3.44%) patients and was treated with analgesics and cold packs. In two (2.29%) patients, vaginal mesh erosion was seen and full recovery was achieved with an excision. The TOT surgical success and complication rates are shown in Table-III.

**Table-III:** Success and surgical complication rates of transobturator tape surgery.

Operative results and complications	n (%)
Full recovery after surgery	77 (88.5)
Urinary retention	0
Perineal pain	3 (3.44)
De novo urge incontinence	5 (5.7)
Mesh erosion	2 (2.29)
Bladder injury	0

## DISCUSSION

Despite the variety of surgical interventions proposed for SUI, tension-free vaginal tape (TVT) continues to be one of the most frequently practiced interventions.^8^ TVT is seen to be as effective as Burch’s colposuspension in two-year follow-up.^9^ Despite its relative safety, potential and major complications such as bladder perforation, and urethral and intestinal injuries have been reported related to the Retzius space. The main concerns regarding these techniques are related to potential vaginal or urethral mesh erosion risks. This is why TOT, which does not decrease the effectiveness in the short term, was designed as an alternative to mesh tapes.^10^ In the transobturator technique defined by Delorme, the tape is passed between the obturator foramina outside-in (tensely towards under the urethra).^3^ Many series have reported similar success rates for the TOT procedure in the treatment of SUI.^9^-^12^,^14^,^16^ In a series in which 117 patients were followed up for three months, DeTayrac reported the one-year recovery rate of TOT as 84% (10). The stress test and urodynamic evaluation performed at the 2^nd^ year in our study showed that TOT had a success rate of 90%. Four (5%) patients who had symptoms of urgency during the follow-up period had de novo urge incontinence. Based on our 2-year experience, it can be said that the TOT demonstrates similar results to the gold standard Burch colposuspension or TVT with its 90% recovery rate. 10,13,14 After a study by Domingo et al. in 2005, the TOT success rate was found as 96.8%.^11^ Grise et al. reported the success rate of the procedure as 80% in their study, which involved following up 206 patients for one year. In addition, they demonstrated that 56.6% and 53.8% of the patients’ immediate urge to urinate with or without urge incontinence disappeared, respectively.^15^ Although rate of newly occurring urge incontinence was 5% and statistically significant in our study, it might also reflect TOT’s minor obstructive effects.

Preoperative and emergency post-operative TOT complications are rare. In the study of Mellier et al., one patient had urethral perforation.^14^ Costa et al. suggested that if the lesion was not superficial, it was safer to delay the implantation.^13^ Bleeding was reported in 0.8% of the patients, which was treated only with compression.^16^,^17^ The perforated lateral urethral space under the pelvic fascia is not crossed by veins. Also, Spinose et al. recently showed that the outside-in pathway was distant from the posterior branch of the obturator nerve and inferior external pudendal artery.^16^ The risk of bladder perforation decreases dramatically with the TOT technique. Krauth et al. calculated this rate to be approximately 0.5%.^17^ No perforation was seen in the routine cystoscopies of 38 patients.^18^ Accordingly, cystoscopy is recommended in cases of previous or simultaneous prolapsus. In our study, a routine cytoscopic procedure was performed on each patient following the surgery and no cases of perforation were seen.

De novo urge incontinence, which is rarely seen after TOT operations, indicates that the obstructive effect of the surgery is minimal. In our study, the rate of de novo urges negatively affecting the quality of life was found as low (5.7%) (n=5), consistent with the literature.^19^,^20^ Perineal pain, which was reported at a rate of 2.3-5% after TOT surgery in the literature, was seen at a rate of 3.44% (n=3) in our study, and disappeared within the first month. The results of our study are consistent with the literature.^21^,^22^ Vaginal mesh erosion was seen in the early period in 2.29% (n=2) of the patients. Our rate is low compared with the review and meta-analysis results by Latthe et al.^23^ Our short follow-up period might account for the low rates of erosion because vaginal erosion is usually one of the long-term complications of TOT surgery.

One of the longest follow-up periods in the literature is in the study by Alcalay et al.^24^ In their study, patients were followed up for 10-20 years and it was determined that the success rate diminished with time and reached a plateau at the end of 10-12 years. Again, in the same study, the success rate was reported as 69%. The newly practiced incontinence surgeries have been compared with this procedure because the long-term results of retropubic colposuspension surgeries are much better known. The success rate of open retropubic colposuspension surgeries has been determined as being between 68.9% and 88%.

Our TOT results emerged as similar to open retropubic colposuspension surgeries.^25^,^26^ However, being less invasive and having fewer adverse effects has made TOT surgery more popular in recent years. Kim et al. compared TOT results at the first and 3^rd^ year and stated that although the success rate was 70% in the first year, it decreased to 60% in the third year. Only our long-term results are given in the present study, which suggests that the first-year results could have been found much higher.^27^

## CONCLUSIONS

Our two-year follow-up results showed that TOT was an effective technique with a low risk of complications. These results support the evidence that the TOT procedure should be considered a better and more beneficial option as compared with TVT.
